# A social determinants of health survey in an Appalachian East Tennessee Medical Center: Initial findings and correlations with physical and emotional states of health

**DOI:** 10.1371/journal.pone.0332087

**Published:** 2025-10-09

**Authors:** Paul D. Terry, Gulsah Onar, Jennifer Ferris, Robert Eric Heidel, Nate Brophy, Kritika Thapa, Laylan Shali, Heidi Worth, Gayathri Kumar, Tracy Walker, Rajiv Dhand

**Affiliations:** 1 Department of Medicine, University of Tennessee Health Science Center College of Medicine, Knoxville, Tennessee, United States of America; 2 Department of Surgery, University of Tennessee Health Science Center College of Medicine, Knoxville, Tennessee, United States of America; 3 Division of Applied Research and Evaluation, Social Work Office of Research and Public Service (SWORPS), University of Tennessee, Knoxville, Tennessee, United States of America; Iran University of Medical Sciences, IRAN, ISLAMIC REPUBLIC OF

## Abstract

**Background:**

People living in Appalachia experience inequities in health outcomes that may result from social determinants of health (SDH) and consequent barriers to healthcare.

**Objective:**

We aimed to assess SDH in our Appalachian patient population and examine associations between SDH and patients’ physical and emotional well-being.

**Methods:**

We constructed and administered a SDH questionnaire in an urban medical center in Appalachian East Tennessee. Our survey included administration of the Short Form-36 (SF-36), which measures various domains of physical and emotional health. We used the SDH questionnaire to enumerate respondents’ barriers to health (a total barrier score), with a maximum of 47 barriers identified. Descriptive statistics were calculated as frequencies and percentages. Spearman’s and Pearson’s (*r*) correlations and hierarchical multiple regression models were used to quantify associations between the SDH barrier scores and SF-36 subscales.

**Results:**

Our patients experienced substantial barriers to health (M = 11.61, SD = 5.10). SDH in our population included being underweight or overweight (BMI < 18.5 or ≥25; 71.2%), having a lower annual family income (<$50,000/year; 60.7%), and experiencing financial difficulty when seeking medical care (51.9%). Some differences in SDH prevalence according to sex were noted, such as a greater proportion of males (12.8%) than females (2.8%) having no health insurance. We observed statistically significant negative correlations between the SDH barriers score and all SF-36 subscales. After controlling for sex, age, and racial group, hierarchical multiple regression models showed a consistent negative relationship between SDH barrier score and all eight SF-36 subscales (*B statistics ranged from −2.04 to −3.66)*.

**Conclusions:**

Patients in our Appalachian population experience a high number of barriers to accessing healthcare that are negatively correlated with measures of physical and emotional well-being. To optimize patient care, assessment of patients’ physical and emotional health should complement the use of SDH questionnaires.

## Introduction

The World Health Organization (WHO) defines social determinants of health (SDH) as the non-medical factors that influence health outcomes, conditions in which people are born, grow, work, live, and age, and the wider set of forces and systems shaping the conditions of daily life [1]. Broad domains of SDH include income, education, employment and job security, food security, housing, early childhood development, social inclusion, and adequate access to health services [1]. Because these factors can have a profound impact on the health and well-being of individuals and communities [1], the assessment of SDH is increasingly common in clinical settings and in population-based studies that seek to better define and quantify the associations driving health inequities [2].

Population-based studies typically assess social determinants of health (SDH) using surveys covering multiple domains [2]. Although no formal guidelines exist, the National Academy of Medicine (NAM) recommended routinely collecting data on 12 social and behavioral factors critical to health outcomes: race/ethnicity, tobacco use, alcohol use, area of residence, neighborhood median household income, educational attainment, financial resource strain, stress, depression, physical activity, social isolation, and intimate partner violence [3]. These data help to inform clinical decision-making, monitor population health, guide health policy, and support research into the causes of health disparities and the impact of interventions [3]. Whereas NAM’s 12 domains provide a universal framework, SDH screening tools are often tailored to specific healthcare or research objectives [4].

Appalachia is a geographic region in the central and southern sections of the Appalachian Mountains [5], including 52 counties in the Appalachian region of eastern and central Tennessee [6]. According to the Appalachian Regional Commission, Appalachia performed worse than the national average in 33 out of 41 health indicators [7,8]. People living in Appalachia face comparatively high barriers to health and healthcare access (henceforward referred to as “barriers to health”) compared with the U.S. as a whole, including a disproportionate occurrence of chronic medical [8,9] and dental [10] diseases, transportation limitations [11,12], vaccine hesitancy [13], low health literacy [14], poverty [8,15], and domestic violence [16], among others, that often lead to inequities in healthcare outcomes. Central Appalachia, which includes areas of East Tennessee, has a median household income 38% below the national median, 16.6% less post-secondary education, and higher-than-average household poverty rates [17]. People affected by low levels of income and education are less likely to have stable and flexible jobs, health insurance, quality medical care, and a healthy lifestyle. Approximately 13.9 percent of Central Appalachia residents receive disability benefits compared with 5.1% of the nation as a whole [17].

Understanding patients’ SDH is critical for reducing healthcare disparities and improving outcomes [18]. This knowledge enables clinicians to tailor treatment plans, connect patients with community resources, and optimize resource allocation [19]. Although prior studies have explored SDH in Appalachian populations [20], few have designed region-specific SDH questionnaires. We developed and administered such a questionnaire at a medical center in East Tennessee, serving a large catchment area across Central and South-Central Appalachia. This survey incorporated the 36-item Short Form survey (SF-36) [21], a validated tool assessing physical and emotional health states.

### Rationale and purpose

Prior SDH screening tools have assessed the type and number of barriers to healthcare access but rarely incorporate individual health status, limiting understanding of how physical and emotional health influence these barriers. For example, patients with robust health may navigate transportation barriers more effectively than those with poor health. In high-barrier regions such as Appalachia, integrating SF-36 assessments of physical and emotional health with SDH screening could enhance understanding of patient needs and guide targeted healthcare strategies. This study has two aims: 1) to characterize SDH and quantify related barriers to healthcare access in an Appalachian patient population; and 2) to examine associations between these barriers and physical and emotional health status measured by the SF-36.

## Materials and methods

### Participants and setting

The target population for this study was adult patients (aged ≥18 years) receiving medical care in hospital settings. The cross-sectional study sample included both outpatients and inpatients who were seen at, or admitted to, the University of Tennessee Medical Center (UTMC), Knoxville, between April 13, 2021, and March 25, 2024. Participants were recruited using a convenience sampling strategy. All participants provided written informed consent prior to completing the survey, and the study protocol was approved by the IRB at UTMC (IRB #4738, approved as Expedited Category 7). All survey responses were de-identified before analysis to ensure participants’ confidentiality.

### Instrumentation

A total of 252 participants completed a structured survey (see S1 Table in the online supporting materials). The survey included seven demographic questions, such as age, place of residence, marital status, sex, and race, along with the SDH and SF-36 questionnaires. The SDH questionnaire included 58 self-reported items distributed across eight domains (**Table 1**), with each item coded as 0 = no barrier and 1 = barrier. For instance, if a participant reported a barrier to health, such as limited access to transportation, the item was coded as 1; otherwise, it was coded as 0. These binary-coded responses were used to calculate the barriers-to-health score for each participant. However, due to skip logic embedded in the questionnaire, not all items were presented to every participant, for instance, some participants were only shown certain items based on their earlier responses. Out of the 58 items in the original questionnaire, only the 47 items that were presented to and completed by all participants were included in the analysis. These 47 items were used to compute each participant’s total number of reported barriers, with possible scores ranging from 0 to 47.

**Table 1:** Instruments and number of items used in the study.

Scale	No. of items
**Social Determinants of Health (SDH)**	
Insurance, Access to Care	6
Access and Adherence to Medicine and Therapies	3
Lifestyle And Access to Necessities	8
Quality of Life	5
Social Support	7
Medical History	15
Occupation, Education and Income	3
**36-Item Short Form Health Survey (SF-36)**	
Physical functioning	10
Role limitation due to physical problems	4
Role limitation due to emotional problems	3
Energy/fatigue	4
Emotional well-being	5
Social functioning	2
Pain	2
General health	5
Health change	1

Note. The full questionnaire is provided in S1 Table in the Appendix.

In addition to the SDH questionnaire, participants completed the 36-Item Short Form Health Survey (SF-36) [21]. The SF-36 was administered to provide context for interpreting participants’ self-reported barriers to health in relation to their physical and mental well-being. The SF-36 comprises eight domains: physical functioning; role limitations due to physical problems; role limitations due to emotional problems; bodily pain; general health perceptions; energy/fatigue; social functioning; and emotional well-being. Each SF-36 item uses categorical response options that were converted to a standardized 0–100 scale, with higher scores indicating better perceived health status. These individual item scores within each domain were averaged to obtain a composite score for that domain.

### Validity

To support the content validity of the SDH score, defined as the extent to which a measure adequately represents the full breadth of the construct [22], relevant literature on healthcare-related barriers was reviewed, and existing measures were examined to inform item development (S2 Table). Additionally, item wording and final selection were guided by experts with clinical and research experience, ensuring the SDH scale captured a comprehensive range of barriers to health. The present study also examined criterion validity, defined as “the extent to which a measure is related to, or explains, a target outcome or criterion” [22]. In an early analysis of data from the first 48 respondents, higher scores on the SDH scale – indicating greater barriers to health – were negatively associated with health-related quality of life, as measured by SF-36 subscales, consistent with expectations [23]. However, because the SDH score is an index composed of formative indicators, where items collectively define the construct, rather than reflect an underlying latent variable, internal consistency metrics such as Cronbach’s alpha are conceptually inappropriate [24] and, therefore, are not reported here.

### Procedures

Convenience sampling was used to recruit 252 participants, including both outpatients and inpatients who received care at the UTMC in Knoxville between April 13, 2021, and March 25, 2024. Each participant was approached in person by study staff and invited to complete a paper-and-pencil survey. Prior to participation, patients were informed about the purpose of the study, their right to decline participation, and their right to withdraw at any time without consequence. Patients were eligible if they were adults and not experiencing a life-threatening medical emergency. Approximately 83.3% of patients who were approached subsequently agreed to participate in the survey. With a two-tailed hypothesis, a hypothesized correlation of 0.2 (small effect) between the SDH and SF-36, an alpha value of 0.05, a power of 80%, and a null hypothesis of no correlation (r = 0.0), a total of n = 193 would be required to achieve adequate statistical power for the study. All completed surveys were entered into SPSS Version 29 (Armonk, NY: IBM Corp.), where they were screened for data entry errors and inconsistencies. Descriptive statistics were computed to summarize participant characteristics prior to running the analysis.

### Data analysis

Descriptive statistics were calculated for all variables. Skewness and kurtosis statistics were computed for the composite SDH score and for each of the SF-36 subscales to assess normality. Spearman’s (non-parametric) and Pearson’s (parametric) correlations were used to quantify associations between SDH and SF-36 scores. Missing data were evaluated and found to be primarily missing by design due to survey skip logic. Nonetheless, the analyses were repeated using multiple imputation, which yielded results that were substantively the same (not shown). Given the similarity in findings, only the results based on the non-imputed data are presented here. Additionally, a series of hierarchical linear regression analyses were conducted to examine the relationship between SDH-Score and the eight domains of the SF-36. For each regression model, age, sex, and racial group were entered as control variables in Step 1, followed by SDH-Score as the primary predictor in Step 2. Age, sex, and racial group were included as covariates to account for variance attributable to demographic characteristics, thereby improving the precision of the estimates and ensuring that observed associations reflected relationships among the primary variables of interest. To account for violations of residual normality and homoskedasticity, all models were bootstrapped with 2,000 resamples to generate robust confidence intervals and standard errors. A Bonferroni correction was implemented to account for the inflated family-wise error rate associated with multiple hypothesis tests, with an adjusted α = 0.006. Lastly, we dichotomized barrier scores and SF-36 scores into “low” vs. “high” based on the median scores, and cross-tabulated the dichotomous variables to examine the concordance of barriers to health and physical and emotional functioning. All analyses were performed using SPSS Version 29 (Armonk, NY: IBM Corp.), R version 4.3.1(Vienna, Austria: R Foundation for Statistical Computing) [25], and RStudio (Boston, MA: Posit Software, PBC) [26].

## Results

### Descriptive statistics

Most of the 252 participants completed the self-administered SDH questionnaire in 15–30 minutes. Respondents’ mean age was 61.7 (*SD* = 16.7) years. The majority were female (56.7%), white/Caucasian (82.5%), single or not living with a partner (50.4%), unemployed, retired, or disabled (62.7%), lived in a rural region (40%), had a high school degree or less education (43.4%), and had a total household income of less than $50,000 (60.8%); however, only 82.9% of respondents provided household income level (**Table 2**).

**Table 2:** Demographic characteristics and female-male differences among participants.

Variable/Level	Total *N* = 252	Female *N* = 143 (56.7%)	Male *N* = 109 (43.3%)	*p*-value
**Mean Age (SD)**	61.7 (16.7)	62.4 (17.8)	60.8 (15.1)	.433
**Mean BMI (SD)**	29.6 (8.0)	30.1 (8.9)	28.8 (6.4)	.181
**Admission Type**				
Inpatient	213 (84.5%)	113 (79.0%)	100 (91.7%)	.01
Outpatient	39 (15.5%)	30 (21.0%)	9 (8.3%)	
**Marital Status**				
Single/ not living with a partner	127 (50.4%)	80 (55.9%)	47 (43.1%)	.059
Married/ living with a partner	125 (49.6%)	63 (44.1%)	62 (56.9%)	
**Race**				
Black/African American	29 (11.5%)	16 (11.2%)	13 (11.9%)	.91
White/Caucasian	207 (82.1%)	119 (83.2%)	88 (80.7%)	
Multiracial or other race	15 (6.0%)	8 (5.6%)	7 (6.4%)	
**Hispanic/Latino**				
No	245 (97.2%)	140 (97.9%)	105 (96.3%)	1
Yes	6 (2.4%)	3 (2.1%)	3 (2.8%)	
**Education**				
High school graduate/GED or less	109 (43.3%)	60 (42.0%)	49 (45.0%)	.163
Post-HS/trade school/some college	84 (33.3%)	43 (30.1%)	41 (37.6%)	
Bachelor’s degree or higher	58 (23.0%)	39 (27.3%)	19 (17.4%)	
**Living environment**				
Urban/Suburban	150 (59.5%)	84 (58.7%)	66 (60.6%)	.894
Rural	100 (39.7%)	57 (39.9%)	43 (39.4%)	
**Has insurance**				
Yes	234 (92.9%)	139 (97.2%)	95 (87.2%)	.002
No	18 (7.1%)	4 (2.8%)	14 (12.8%)	
**Medicare insurance coverage**				
Yes	142 (56.3%)	93 (65.0%)	49 (45.0%)	.029
No	89 (35.3%)	45 (31.5%)	44 (40.4%)	
**Medicaid insurance coverage**				
Yes	50 (19.8%)	32 (22.4%)	18 (16.5%)	.514
No	181 (71.8%)	106 (74.1%)	75 (68.8%)	
**Insurance type**				
Public	178 (70.6%)	114 (79.7%)	64 (58.7%)	.015
Private	53 (21.0%)	24 (16.8%)	29 (26.6%)	
**Employment status**				
Employed	91 (36.1%)	43 (3.1%)	48 (44.0%)	.076
Unemployed/Homemaker	33 (13.1%)	19 (13.3%)	14 (12.8%)	
Retired/Disabled	120 (47.6%)	75 (52.4%)	45 (41.3%)	
**Household income**				
Less than $50,000	127 (5.4%)	74 (51.7%)	53 (48.6%)	.66
$50,000 or more	82 (32.5%)	45 (31.5%)	37 (33.9%)	
**Time since last routine check-up**				
Within 2 years or less	213 (84.5%)	125 (87.4%)	88 (80.7%)	.109
More than 2 years ago	37 (14.7%)	16 (11.2%)	21 (19.3%)	
**Barriers to seeing a doctor (past 2 years)** ^**a**^				
Money or finance	28 (11.1%)	14 (9.8%)	14 (12.8%)	.481
Did not have transportation	12 (4.8%)	8 (5.6%)	4 (3.7%)	.536
Work schedule	4 (1.6%)	1 (0.7%)	3 (2.8%)	.308
Long distance	9 (3.6%)	5 (3.5%)	4 (3.7%)	1
Child or Dependent Care	1 (0.4%)	1 (0.4%)	0 (0.0%)	1

P-values are examining male-female differences. For continuous variables, p-values report independent samples t-tests; for categorical variables, they report chi-squared or Fisher’s exact tests.

^a^Frequencies and proportions of “No” responses suppressed for brevity.

**Fig 1:**
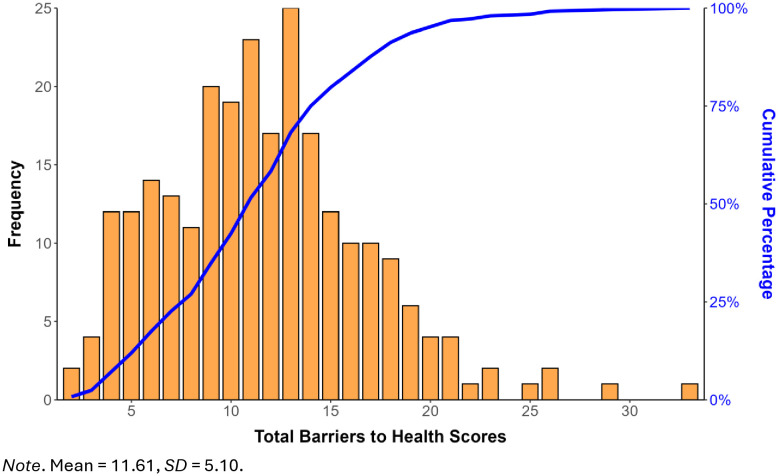
Frequency and cumulative distribution of total barriers to health scores. The figure displays the frequency distribution (histogram) and cumulative distribution curve (blue line) of Total Barriers to Health Scores.

Participants reported a considerable number of barriers to health (*M *= 11.58, *SD* = 5.11, range 3–33); **Fig 1**), with no patient reporting fewer than two barriers. The right-skewed distribution of barrier scores shows that whereas many participants reported multiple barriers to health, few reported more than 30 barriers, and the maximum score of 47 was not reported by any participant. The five most prevalent social determinants of health were BMI outside the normal range (BMI < 18.5 or > 25; 71.8%), presence of unhealthy lifestyle behaviors (e.g., physical inactivity; 63.5%), low household income (60.8%), hypertension (59.5%), and structural barriers to healthcare access (e.g., financial; 54.3%). Conversely, the five least prevalent determinants were lack of access to clean water (0.8%), intimate partner safety concerns (1.8%), inadequate access to communication equipment (2.8%), homelessness (4.4%), and absence of close social support (5.6%).

We observed some differences in the barriers patients experienced according to sex; for example, a greater proportion of males (12.8%) than females (2.8%) reported having no health insurance (**Table 2**). Approximately 14% of respondents did not see a doctor or healthcare professional regularly, and approximately 15% had not received a routine checkup in over 2 years. Males (19.3%) were less likely to have had a routine medical check-up in the past two years than females (11.2%), although the latter difference was not statistically significant (p = 0.11). In subanalyses (data not shown), participants who did not have regular follow-up with their doctor reported consuming a greater number of cigarettes, lower uptake of flu and/or COVID vaccines, and higher experience of emotional problems than patients who had regular visits with their doctor. Significant proportions of respondents who did not have routine checkups cited financial strain (38.9%) and distance/ lack of transportation (29.2%) as reasons. Financial reasons (21.3%) were also cited by respondents for not taking their prescribed medicines or therapies.

### Bivariate correlations

We observed statistically significant negative correlations between the barriers score and all eight SF-36 subscales: Physical Functioning (*r* = −0.52, *p* < 0.001), Role Limitations Due to Physical Health (*r* = −0.47, *p* < 0.001), Role Limitations Due to Emotional Health (*r* = −0.42, p < 0.001), Energy/Fatigue (*r* = −0.47, *p* < 0.001), Emotional Wellbeing (*r* = −0.47, *p* < 0.001), Social Functioning (*r* = −0.45, p < 0.001), Pain (*r* = −0.42, p < .001), and General Health (*r* = −0.43, p < 0.001) (**Table 3**). Barrier scores ranged between 2–33, and all SF-36 subscales ranged between 0–100. When barrier scores and SF-36 scores were dichotomized into “high” and “low” and cross-tabulated, approximately 26% of participants (n = 66) had “low” physical and/or emotional functioning scores despite having “low” barrier scores (data not shown). Likewise, approximately 6% of participants (n = 15) had “high” physical and/or emotional functioning despite “high” barrier scores. Approximately 23% of participants had both “high” levels of functioning and “low” barriers scores, whereas approximately 44% of participants had “low” levels of functioning in addition to “high” barrier scores.

**Table 3:** Correlations between quality of life and demographic and SDH scores in Appalachian patients.

Variable	*M*	*SD*	1	2	3	4	5	6	7	8	9	10	11
**Controls**													
1. Age	61.69	16.88											
2. Sex	.42	.50	−.05										
3. Racial Group	.18	.38	−.18^**^	.02									
**Predictor**													
4. SDH Score	11.58	5.11	.05	−.02	.02								
**Outcomes (SF-36)**													
5. Physical Functioning	45.65	32.89	−.29^***^	.05	.04	−.52^***^							
6. RF-Physical	27.35	39.69	−.16^*^	.12	.13^*^	−.47^***^	.61^***^						
7. RF-Emotional	69.66	41.82	.13^*^	.02	.00	−.42^***^	.28^***^	.34^***^					
8. Energy/fatigue	35.00	25.76	−.10	.13	.09	−.47^***^	.49^***^	.51^***^	.32^***^				
9. Emotional Well-being	70.74	22.67	.14^*^	.09	−.07	−.47^***^	.28^***^	.33^***^	.54^***^	.45^***^			
10. Social Functioning	67.50	29.02	.01	.07	.05	−.45^***^	.41^***^	.48^***^	.54^***^	.47^***^	.60^***^		
11. Pain	43.79	30.37	.09	.00	.08	−.42^***^	.37^***^	.42^***^	.31^***^	.37^***^	.30^***^	.47^***^	
12. General Health	47.59	24.50	−.09	.13^*^	.07	−.43^***^	.53^***^	.47^***^	.29^***^	.57^***^	.38^***^	.42^***^	.30^***^

N = 245. RF = Role Functioning; *M* = Mean; *SD* = Standard Deviation. Participants ranged in age between 18–35; Social Determinants of Health (SDH) scores ranged between 2–33; all Short Form Health Survey (SF-36) subscales ranged between 0–100. The correlation between Sex (female = 0; male = 1) and Racial Group (white = 0; non-white = 1) is a phi coefficient. The correlations between dichotomous and continuous variables are point-biserial correlations. The remaining tests are Pearson correlation coefficients. Row numbers index the variables and correspond to the numbered columns.

*p < .05, **p < .001, ***p < .001.

### Hierarchical multiple regression models

The demographic variables alone (Step 1) explained between 1% and 9% of the variance in SF-36 subscales. After controlling for these demographic factors, the addition of SDH-Score in Step 2 significantly improved prediction across all health domains, accounting for an additional 18% to 26% of the variance (**Table 4**). The *F*-change values (range: 55.05–96.52, all *p* < 0.001) indicated that the addition of SDH-Score significantly improved model fit across all health domains. Moreover, all associations remained statistically significant even after applying the conservative Bonferroni correction for multiple comparisons.

**Table 4:** Hierarchical linear regressions for SDH-score predicting SF-36 subscales.

Outcome	Intercept	*B* (*SE*)	95% CIs	β (*SE*)	Δ*F*	*R* ^2^		
						Step 1	Step 2	Δ
Physical Functioning	115.58	−3.29^*^ (.35)	[−4.01, −2.63]	−.51^*^ (.05)	96.52^*^	.09	.34	.26
Role Functioning-Physical	80.20	−3.66^*^ (.48)	[−4.64, −2.75]	−.46^*^ (.06)	73.51^*^	.05	.27	.22
Role Functioning-Emotional	85.42	−3.52^*^ (.43)	[−4.38, −2.65]	−.42^*^ (.05)	56.07^*^	.02	.20	.18
Energy/fatigue	64.13	−2.38^*^ (.27)	[−2.91, −1.86]	−.46^*^ (.05)	71.59^*^	.03	.25	.22
Emotional Well-being	80.71	−2.13^*^ (.23)	[−2.57, −1.68]	−.47^*^ (.05)	75.03^*^	.03	.26	.23
Social Functioning	90.60	−2.60^*^ (.35)	[−3.33, −1.92]	−.45^*^ (.06)	64.10^*^	.01	.21	.20
Pain	57.93	−2.57^*^ (.34)	[−3.25, −1.93]	−.43^*^ (.06)	56.42^*^	.02	.20	.18
General Health	72.72	−2.04^*^ (.29)	[−2.62, −1.47]	−.42^*^ (.06)	55.05^*^	.03	.21	.18

Age, sex (male; female), and racial group (white; nonwhite) were entered as controls in Step 1, with SDH-Score added as the predictor in Step 2. All models were bootstrapped with 2,000 resamples to generate confidence intervals and standard errors robust to violations of residual normality and homoskedasticity.

*p < .001. Adjusted p-values for all regression coefficients remained statistically significant after applying Bonferroni correction (adjusted α = .006).

SDH-Score demonstrated strong negative associations with all SF-36 subscales. The strongest relationship was observed with Role Functioning-Physical (*B* = −3.66, *p* < 0.001), indicating that an increase in reported barriers to health was associated with significantly poorer role functioning due to physical problems. Similarly substantial negative associations were found with Role Functioning-Emotional (*B* = −3.52, *p* < 0.001) and Physical Functioning (*B* = −3.29, *p* < 0.001). The remaining health domains also showed consistent negative relationships with SDH-Score: Social Functioning (*B* = −2.60, *p* < 0.001), Pain (*B* = −2.57, *p* < 0.001), Energy/fatigue (*B* = −2.38, *p* < 0.001), Emotional Well-being (*B* = −2.13, *p* < 0.001), and General Health (*B* = −2.04, *p* < 0.001).

## Discussion

We developed and administered a novel SDH questionnaire to 252 adult inpatients and outpatients at the University of Tennessee Medical Center (UTMC) in Appalachian East Tennessee. As in previous studies [4], we balanced NAM recommended measures with items addressing local needs. Barriers to health in our population were similar to those documented in other Appalachian groups [8,12,15,17], including lower educational attainment and household income compared with the U.S. general population [27,28]. Calculating a barrier score provided a quantitative assessment of healthcare access difficulties, information that can guide targeted interventions in underserved communities with high barrier scores.

In their 2017 publication, Billioux et al. [29] reviewed over 50 screening tools for SDH but did not list them. Instead of replicating their search, we reference S2 Table, which includes four key U.S. SDH screening tools identified by other authors [30] for primary care settings: (1) the Protocol for Responding to and Assessing Patients’ Assets, Risks, and Experiences (PRAPARE) tool by the National Association of Community Health Centers [31,32], (2) the Accountable Health Communities Screening Tool [29,33], (3) the Health Leads Screening Tool [34], and (4) the HealthBegins Upstream Risks Screening Tool [35]. S2 Table also includes six additional studies [36–41] cited as relevant screening tools. These 10 studies provide a contemporary sample for comparing key aspects of our own tool. Unlike most SDH tools, which focus solely on healthcare access barriers, our tool also assesses individual health states, such as the SF-36 domains of physical functioning, general health, physical and emotional roles, and vitality. This enables providers and researchers to better understand how health status influences a patient’s ability to overcome barriers, such as transportation challenges, which a healthier patients may navigate more easily that one with poor physical or emotional health.

The association between health barriers and patients’ health is complex and not unidirectional. For example, barriers to health can adversely affect physical and emotional health [18,19], which themselves can lower patients’ access to care. Whereas the SF-36 measures of physical and emotional health showed moderate inverse correlations with the reported number of barriers, some patients with few barriers still reported low physical or emotional functioning. Conversely, some patients maintained high functioning despite facing many barriers. How and why patients with higher functioning might better overcoming specific barriers compared to those with lower functioning remains understudied.

Understanding patients’ physical and emotional states of health is key to reduce health disparities, particularly when using SDH data. We found that nearly half of participants had low physical and emotional functioning, along with significant barriers to care. This group requires more focused support to improve their well-being and access to health services. Locally, for example, future studies should examine the ways in which SDH may interact with individuals’ physical and emotional well-being to produce health disparities. Given the observed interconnections between several SDH, such as a lower vaccine uptake among patients who did not regularly visit their primary care physician, and lower likelihood of receiving routine checkups among those with limited access to transportation, our future efforts will focus on clarifying these multiple connections among SDH and states of health to promote public health. In addition, because our results show important sex differences, for example, women are more likely to use of health insurance and primary care services, we will also explore sex-specific connections between SDH and well-being in our ongoing analyses.

Our study was designed to better understand the barriers to achieving optimal benefit from healthcare in our patient population, rather than the more typical aim of integrating SDH into electronic health records (EHR) (S2 Table). Consequently, our 65-item SDH questionnaire is considerably longer than the 11-item to 32-item range reported for several previous instruments (S1 Table). Although our questionnaire overlaps substantially with others in the SDH domains covered, our instrument assessed most domains with greater granularity and, unlike most others, included questions about patients’ medical histories, which will be helpful in identifying and addressing barriers specific to certain subgroups. In future analyses, we intend to examine these domains in detail to better understand which SDH have the greatest impact on those seeking healthcare in our Appalachian environment. Integrating data from our questionnaire into EHR will involve a subset of questions, with the selection informed by patients’ responses to the broader array used in this research phase.

SDH screening tools are often tailored to the needs of specific populations or research questions, with the number of questions and domains a compromise between obtaining sufficient data and minimizing burdens on respondents and staff (S2 Table). NAM’s 12 domains of SDH [3] may seem too extensive for some clinics, for example, in which case Healthy People 2030’s five domains: (1) economic stability, (2) education access and quality, (3) health care access and quality, (4) neighborhood and built environment, and (5) social and community context [42], may be more suitable. Like NAM’s framework [3], these have been used to guide SDH data collection and categorization in both patient screening [20,43] and research [44]. Despite its length, respondents typically completed our survey within 15–30 minutes and did not appear unduly burdened. Therefore, it may provide a useful model for investigators designing surveys in populations with similar health disparities.

Our study has several limitations. Although participation in our survey was relatively high (~83.3%), it is possible that non-respondents had unique associations between barriers to health and individual states of physical and emotional health, introducing bias. Similarly, our convenience sample included only patients who sought care at our institution, were healthy enough to participate, and were approached during clinic hours; respondents may therefore not represent of all inpatients and outpatients at UTMC during the study period. In addition, respondents who overcame barriers to become patients at UTMC may differ from those who did not seek care, or who sought care elsewhere, and for whom barriers may be greater. Although Appalachian populations have shared cultural features and influences, including health-seeking behavior [45], our survey population may not be representative of patient populations in other regions of Appalachia. Our cross-sectional design also limits causal inference; prospectively collected data could better demonstrate whether barriers to health predicts sub-optimal care. It is also possible that patients in poor health were more likely to report certain SDH than healthier patients, which would bias correlations with SF-36 measures. Finally, we quantified respondents’ barriers using a simple additive method that assumes equal weight and severity across barriers. Although interpretable, this method may oversimplify participants’ lived experiences; weighted or severity-adjusted models could better capture the nuanced and varying impact of individual barriers.

## Conclusion

Patients in our Appalachian population experience face numerous barriers to healthcare, such as limited transportation and financial resources. These barriers are negatively correlated with physical and emotional well-being, yet the latter are often overlooked in efforts to improve patient health using SDH screening tools. To optimize care in regions with significant barriers to healthcare, like our Appalachian community, strategies should incorporate data on patients’ states of physical and emotional health alongside SDH questionnaires.

## Supporting information

S1 Table The Social Determinant of Health Survey used in data collection in 2024. (DOCX)

S2 Table Summary of domains and dimensions in the social determinants of health (SDH) questionnaire. (DOCX)

S3 Table Some well-known previous attempts to develop and implement social determinants of health questionnaires. (DOCX)

S1 File Dataset – de-identified. (XLSX)
