# Supplementary material for: A social determinants of health survey in an Appalachian East Tennessee Medical Center: Initial findings and correlations with physical and emotional states of health
Source: PLoS One. 2025 Oct 9;20(10):e0332087. doi: 10.1371/journal.pone.0332087 (PMC12510578; doi:10.1371/journal.pone.0332087)
Supplement: S1 Table — (DOCX) [file pone.0332087.s001.docx]

**S1 Table. The Social Determinant of Health Survey used in data collection in 2024.**

**Social Determinants of Health Interview**

# INTRODUCTION

**Thank you for agreeing to take part in this study. During this interview I will be asking you questions about your background and experiences with your health and healthcare. If any of the information is hard to recall, please give me your best guess. If we come to any question that you don’t want to answer, let me know. We will skip it. There are no right or wrong answers to any of the questions. I want to hear about your experience. Please keep in mind that your taking part is voluntary. You are free to stop this interview at any point.**

**Do you have any questions before we start?**

**We would like to know your home address. Your address is to learn more about your neighborhood and will be kept strictly confidential. Your address itself will never be disclosed and will never be used in our research study.**

**Respondent’s home address**

Participant’s study number: ___ ___ ___

Participant’s initials: ___ ___ ___

Date of Interview: ___ ___ /___ ___ /___ ___ ___ ___

Month Day Year

Time Interview Began: ___ ___: ___ ___ AM or PM

Hour Minute

Time Interview Ended: ___ ___: ___ ___ AM or PM

Hour Minute

INPATIENT or OUTPATIENT ____________

| **The Notice of Research was presented to the respondent; the respondent’s concerns and questions were** | |
| --- | --- |
| **addressed.** |  |

**Interviewer ID: ___ ___ ___**

----------------------------------------------------------

# DEMOGRAPHICS

**A1.** What is your year of birth?

___ ___ ___ ___

Year

**A2.** Where were you born?

If U.S. born, ______________________, ___________________

City/Town State

If not US born, ____________________, ___________________

City/Town Country

Citizenship at birth ______________________

Country

**A3.** What city and state do you live in?

______________________, ___________________

City/Town State

**A4.** What is your current marital status?

1.  Single, never married
2.  Married
3.  Living with partner
4.  Divorced or separated
5.  Widowed

9  NA

**A5.** Which of these settings best describes where you live?

1.  City / Urban
2.  Country / Rural
3.  Suburbs (near a city)
4.  Other, specify ______________________________

9  NA

**A6.** How would you describe yourself in terms of gender?

1.  Female
2.  Male
3.  Other, specify ______________________________

**A7.** How would you describe yourself in terms of race/ethnicity?

1.  American Indian or Alaska Native
2.  Asian
3.  Black or African American
4.  Hispanic or Latino
5.  Native Hawaiian or Other Pacific Islander
6.  White
7.  Multiracial or Other, specify ______________________________

9  NA

----------------------------------------------------------

# B. INSURANCE, ACCESS TO CARE

Now I’m going to ask you some questions about your health care and insurance.

**B1.** Do you have health insurance or some form of health care coverage right now?

1.  Yes

**IF NO, GO TO B3**

1.  No

9  NA

## Code No=1; Code Yes=0; Code 9= “.”

**B2.** What type of health insurance coverage do you have? Check all that apply.

1.  Insurance provided by employer (yours or spouses)
2.  Insurance paid for fully by you
3.  Medicare
4.  Supplemental Medicare insurance
5.  Medicaid
6.  Other, specify _________________________________

9  NA

**B3**. Think about the past year. Did you go to any of these places when you needed medical care?

(Check all that apply.)

1.  A hospital emergency room
2.  A health department or community health clinic
3.  An urgent care or walk-in clinic
4.  A hospital outpatient clinic
5.  A private doctor’s office
6.  Some other place, specify ______________________________
7.  Did not go somewhere for medical care

**GO TO B5**

# 9  NA

**B4**. Which place did you go to most often for medical care?

1.  A hospital emergency room
2.  A health department or community health clinic
3.  An urgent care or walk-in clinic
4.  A hospital outpatient clinic
5.  A private doctor’s office
6.  Some other place, specify ______________________________

9  NA

## Code 1=1; Code 2-6=0; Code 9= “.”

**B5.** Do you have a person that you think of as your regular doctor or health care provider?

1.  Yes
2.  No

9  NA

## Code 1=0; Code 2=1; Code 9= “.”

**B6**. About how long has it been since you last visited a doctor for a routine checkup?

A routine checkup is a physical exam or wellness visit. It is not an exam for an injury, illness, or condition.

1.  Within the past year (less than 12 months ago)
2.  Within the past 2 years (more than 1 year but less than 2 years ago)
3.  Within the past 5 years (more than 2 years but less than 5 years ago)
4.  5 or more years ago
5.  Never

9  NA

## Code 1-2=0; Code 3-5=1 Code 9= “.”

**B7**. During the past two years, was there ever a time when you wanted to see a doctor but could not?

1.  Yes
2.  No

**GO TO C1**

9  NA

## Code 1=1; Code 2=0; Code 9= “.”

**B8**. What were the reasons that you were not able to see a doctor? Check all that apply.

1.  Money or finances
2.  Did not have transportation
3.  The distance was too far
4.  Physical/emotional health problems
5.  Domestic violence
6.  Lack of trust in doctors/ healthcare professionals/healthcare
7.  Work schedule
8.  Child or dependent care
9.  Other, specify __________________________________

99  NA

## Code 1-6=1; Code 7-9=0; Code 99= “.” (Note: “Other” responses should be carefully assessed to determine if there are underlying reasons that could present barriers)

----------------------------------------------------------

### C. ACCESS AND ADHERENCE TO MEDICINE AND THERAPIES

Now I’m going to ask you some questions about your medicines and treatments.

**C1.** Are you currently prescribed medicines or treatments by your doctor or healthcare provider?

1.  Yes
2.  No

**SKIP TO C4**

# 9  NA

**C2.** Do you ever have trouble taking your medicine or therapy as prescribed?

1.  Yes

**SKIP TO C4**

1.  No

9  NA

## Code 1=1; Code 2=0; Code 9= “.”

**C3**. What were the reasons that you were not able to take your medicine or therapy? Check all that apply.

1.  Money or finances
2.  The medicine or therapy doesn’t work well enough
3.  I’m taking too many medicines
4.  Contacting a pharmacist is too much trouble
5.  I do not have transportation
6.  Taking the medicine or therapy is too hard
7.  I don’t like the side effects of the medicine or treatment
8.  I forget to take the medicine or therapy
9.  My work schedule gets in the way of taking my medicine or therapy
10.  I don’t want other people to know I’m taking this medicine or therapy
11.  Other, specify __________________________________
12.  NA

**C4.** Did you get a flu shot (flu vaccine) this year?

**SKIP TO C6**

1  Yes

2  No

9  NA

## Code 1=0; Code 2=1; Code 9= “.”

**C5.** What are the reasons that you didn’t get a flu shot?

1.  I don’t trust it to work
2.  I don’t trust it to be safe
3.  I will most likely not get the flu
4.  Getting the flu doesn’t bother me
5.  I am afraid of needles
6.  It’s too much trouble to get the vaccine
7.  The flu shot makes me feel sick
8.  Other, specify __________________________________
9.  NA

**C6.** Did you take COVID-19 vaccine?

**SKIP TO D1**

1.  Yes
2.  No

9  NA

## Code 1=0; Code 2=1; Code 9= “.

**C7.** What were the reasons that you didn’t take a COVID-19 vaccine?

1.  I don’t trust the vaccine to work
2.  I am concerned that the vaccine will make me sick
3.  I am concerned that the vaccine has not been tested well enough
4.  Getting COVID-19 doesn’t bother me
5.  I am afraid of needles
6.  It’s too much trouble to get the vaccine
7.  I believe that I already had COVID-19 and am immune to it
8.  Other, specify __________________________________
9.  NA

----------------------------------------------------------

### D. LIFESTYLE AND ACCESS TO NECESSITIES

Now I’m going to ask you some questions about your lifestyle and access to food and other items.

**D1.** Do you ever have trouble getting enough food?

1.  Yes
2.  No

**SKIP TO D3**

9  NA

## Code 1=1; Code 2=0; Code 9= “.”

**D2**. What are the reasons that you have trouble getting enough food? Check all that apply.

1.  Money or finances
2.  Lack of transportation to grocery store
3.  No grocery store nearby
4.  Health reasons prevent me from shopping
5.  My work schedule prevents me from shopping
6.  Health reasons prevent me from eating enough
7.  Homelessness
8.  Other, specify __________________________________
9.  NA

**D3.** Do you ever have trouble getting fresh, clean water?

1.  Yes
2.  No

**SKIP TO D5**

9  NA

## Code 1=1; Code 2=0; Code 9= “.”

**D4**. What are the reasons that you have trouble getting fresh, clean water? Check all that apply.

1.  Lack of water facilities at home
2.  Unclean water facilities at home
3.  Homelessness
4.  Other, specify__________________________________

9  NA

**D5.** Do you do any of these activities often? Check all that apply.

1.  Smoke tobacco
2.  Drink alcohol
3.  Exercise
4.  Eat fruit and vegetables

9  NA

## Code 1-2=1; Code 3-4=0; Code 9= “.” (Note: Smoking and drinking often, NOT exercising often, NOT eating fruit and vegetables often are barriers and should be coded as 1).

**D6.** Are you experiencing homelessness?

**SKIP TO D8**

### 1  Yes

2  No

9  NA

## Code 1=1; Code 2=0; Code 9= “.”

**D7.** Which of these describes your home?

1.  I own my home
2.  I rent my home
3.  I am staying with family members
4.  I live with friends

9  NA

## Code 3,4=1; Code 1,2=0; Code 9= “.”

**D8.** Do you feel safe in your neighborhood?

1.  Yes, I feel safe in my neighborhood
2.  No, I do not feel safe in my neighborhood

9  NA

## Code 2=1; Code 1=0; Code 9= “.”

**D9.** Do you have any of these services? Check all that apply.

1.  Landline (telephone service)
2.  Cell phone
3.  Internet
4.  None of the above

9  NA

## Code 4=1; Code 1-3=0; Code 9= “.”

**D10.** Do you drive?

1.  I have a driver’s license and am able to drive
2.  I do not have a driver’s license  **SKIP TO D12**
3.  I have a driver’s license but am not able to drive right now

9  NA

## Code 2,3=1; Code 1=0; Code 9= “.”

**D11.** Do you own or have access to a car?

1.  I own a car
2.  I don’t own a car or I do not have access to a car
3.  I don’t own a car, but I have access to cars that I can drive when I need to

9  NA

**D12.** Do you need or use transportation assistance? (Check all that apply)

1.  Yes, my family/friends help me as needed
2.  Yes, I use public transportation services as needed
3.  Yes, I need transportation assistance, but I lack anyone to assist me.
4.  Yes, I need transportation, but the services in my area are unreliable.
5.  No, I do not need transportation assistance.

9  NA

----------------------------------------------------------

# E. QUALITY OF LIFE

**These questions ask for your views about your health during the past 4 weeks.**

**E1.** Overall, how would you rate your health during the **past 4 weeks?**

1.  Excellent
2.  Very good
3.  Good
4.  Fair
5.  Poor

9  NA

## Code 5=1; Code 1-4=0; Code 9= “.”

**E2.** Think about the **past 4 weeks**. How much did physical health problems limit your normal movements, such as walking or climbing stairs?

1.  Not at all
2.  Very little
3.  Somewhat
4.  Quite a lot
5.  Could not do physical activities

9  NA

## Code 4,5=1; Code 1-3=0; Code 9= “.”

**E3.** During the **past 4 weeks**, how hard was it for you to do your daily work because of your physical health?

1.  Not at all
2.  Very little
3.  Somewhat
4.  Quite a lot
5.  Could not do daily work

9  NA

## Code 4,5=1; Code 1-3=0; Code 9= “.”

**E4.** Think about the **past 4 weeks.** How much have you been bothered by **emotional problems**, such as feeling anxious, stressed, depressed or irritable?

1.  Not at all
2.  Slightly
3.  Moderately
4.  Quite a lot
5.  Extremely

9  NA

## Code 4-5=1; Code 1-3=0; Code 9= “.”

**E5.** During the **past 4 weeks**, how much did your physical health or emotional problems limit your normal social activities with family or friends?

1.  Not at all
2.  Very little
3.  Somewhat
4.  Quite a lot
5.  Could not do activities

9  NA

Code 4-5=1; Code 1-3=0; Code 9= “.”

----------------------------------------------------------

# F. SOCIAL SUPPORT

The below questions are about who you can turn to when you need help or support. For each statement, please tell me whether you strongly agree, agree, neither agree nor disagree, disagree, or strongly disagree.

**F1.** There is a special person around me when I am in need. 1  Strongly disagree

1.  Disagree
2.  Neither agree nor disagree
3.  Agree
4.  Strongly agree

9  NA

## Code 1-2=1; Code 3-5=0; Code 9= “.”

**F2.** There is a special person with whom I can share my joys and sorrows.

1.  Strongly disagree
2.  Disagree
3.  Neither agree nor disagree
4.  Agree
5.  Strongly agree

9  NA

## Code 1-2=1; Code 3-5=0; Code 9= “.”

**F3.** Do you feel physically and emotionally safe in your relationship?

1.  Yes, I feel safe in my relationship with my spouse or living partner
2.  No, I do not feel safe in my relationship with my spouse or living partner 9  NA

## Code 2=1; Code 1=0; Code 9= “.”

**F4.** I get the emotional help and support I need from my family.

1.  Strongly disagree
2.  Disagree
3.  Neither agree nor disagree
4.  Agree
5.  Strongly agree

9  NA

## Code 1-2=1; Code 3-5=0; Code 9= “.”

**F5.** I can count on my friends when things go wrong. 1  Strongly disagree

1.  Disagree
2.  Neither agree nor disagree
3.  Agree
4.  Strongly agree

9  NA

## Code 1-2=1; Code 3-5=0; Code 9= “.”

**F6.** I have friends with whom I can share my joys and sorrows. 1  Strongly disagree

1.  Disagree
2.  Neither agree nor disagree
3.  Agree
4.  Strongly agree

9  NA

## Code 1-2=1; Code 3-5=0; Code 9= “.”

**F7.** My family is willing to help me make decisions. 1  Strongly disagree

1.  Disagree
2.  Neither agree nor disagree
3.  Agree
4.  Strongly agree

9  NA

Code 1-2=1; Code 3-5=0; Code 9= “.”

----------------------------------------------------------

# G. MEDICAL HISTORY

Below are a few questions about your height, weight, and medical history.

**G1.** What is your current height (in)? ________________

**G2.** What is your current weight (lb)? ________________

## Code BMI > 25 or < 18.5 =1; code BMI 18.5-24.99=0; code missing = “.”

| Have you ever been told by a doctor or health professional that you have any of these health conditions? | |  | | How old were you when you were diagnosed with this? | Cicle if it is ongoing or resolved issue |
| --- | --- | --- | --- | --- | --- |
| **G3.** | Asthma or COPD? | G3a. | 1.  Yes → 2.  No   9  NA | G3b. __ __ years **99** = NA | 1. Ongoing  2. Resolved |
| **G4.** | Arthritis or rheumatism? | G4a. | 1.  Yes → 2.  No   9  NA | G4b. __ __ years **99** = NA | 1. Ongoing  2. Resolved |
| **G5.** | Diabetes? | G5a. | 1.  Yes → 2.  No   9  NA | G5b. __ __ years **99** = NA | 1. Ongoing  2. Resolved |
| **G6.** | Digestive problems such as ulcer, colitis, or gallbladder disease? | G6a. | 1.  Yes → 2.  No   9  NA | G6b. ___ __ years **99** = NA | 1. Ongoing  2. Resolved |
| **G7.** | Heart trouble such as angina, congestive heart failure, coronary heart disease, or heart attack? | G7a. | 1.  Yes → 2.  No   9  NA | G7b. __ __ years **99** = NA | 1. Ongoing  2. Resolved |
| **G8.** | Psychiatric/emotional illness? | G8a. | 1.  Yes → 2.  No   9  NA | G8b. __ __ years **99** = NA | 1. Ongoing  2. Resolved |
| **G9.** Kidney disease? | | G9a. | 1.  Yes → 2.  No   9  NA | G9b. __ __ years **99** = NA | 1. Ongoing  2. Resolved |
| **G10.** Liver problems? | | G10a. 1  Yes →  2  No  9  NA | | G10b. __ __ years **99** = NA | 1. Ongoing  2. Resolved |
| **G11.** Stroke? | | G11a. 1  Yes →  2  No  9  NA | | G11b. ___ __ years **99** = NA | 1. Ongoing  2. Resolved |
| **G12.** High blood pressure? | | G12a. 1  Yes →  2  No  9  NA | | G12b. __ __ years **99** = NA | 1. Ongoing  2. Resolved |
| **G13.** High cholesterol? | | G13a. 1  Yes →  2  No  9  NA | | G13b. __ __ years **99** = NA | 1. Ongoing  2. Resolved |
| **G14.** Anemia? | | G14a. 1  Yes →  2  No | | G14b. __ __ years **99** = NA | 1. Ongoing  2. Resolved |
|  |  | 9  NA | |  |  |
| **G15.** Osteoporosis? | | G15a. 1  Yes →  2  No  9  NA | | G15b. __ __ years **99** = NA | 1. Ongoing  2. Resolved |
| **G16.** Cancer? | | G16a. 1  Yes →  2  No  9  NA | | G16b. __ __ years  **99** = NA | 1. Ongoing  2. Resolved |

Code 1=1; Code 2=0; Code 9= “.”

----------------------------------------------------------

H. OCCUPATION, EDUCATION AND INCOME

These are the last few questions on the survey.

**H1.** What is the highest level of school that you have finished?

1.  8^th^ grade or less
2.  9^th^ to 11^th^ grade
3.  High school graduate or GED
4.  Post high school trade or technical school
5.  Some college, but no degree
6.  Associate’s degree
7.  Bachelor’s degree
8.  Graduate or professional degree
9.  NA

## Code 1-3=1; Code 4-8=0; Code 9= “.”

**H2.** The next questions are about your current job and the job you have done the longest during your life. These jobs can include any work outside the home. It can also include working on a family farm, running a business from your home, or being a homemaker.

**[WRITE IN JOB TITLE AND (GENERAL) WORKPLACE.**

| What is the job title for your current job? | For what kind of business or industry do you work? | How long have you had this job? |
| --- | --- | --- |
| **__________________________** | **_________________________** | . ___ ___ years |
| What is the job title for the job that you have done the longest during your life? | For what kind of business or industry did you work? | How long did you have this job? |
| **__________________________** | **_________________________** | ___ ___ years |

If your longest held job is the same as your current job, record “same.”

**H3.** Which category best describes your total family income for last year, before taxes?

1.  Less than $10,000
2.  $10,000 to $24,999
3.  $25,000 to $49,999
4.  $50,000 to $74,999
5.  $75,000 to $100,000
6.  More than $100,000

9  NA

## Code 1-3=1; Code 4-6=0; Code 9= “.”

**H4.** Do you or anyone in your family receive public benefits, such as SNAP or food stamps, WIC benefits (Women, Infants, Children), CHIP (Children’s Health insurance Program), housing assistance such as housing voucher or public housing, SSI (Supplemental Security Income), SSDI (Social Security Disability Income) or any local, state, or government-sponsored assistance?

1.  Yes
2.  No

## 9  NA

## Code 1=1; Code 2=0; Code 9= “.”
