# Supplementary material for: A social determinants of health survey in an Appalachian East Tennessee Medical Center: Initial findings and correlations with physical and emotional states of health
Source: PLoS One. 2025 Oct 9;20(10):e0332087. doi: 10.1371/journal.pone.0332087 (PMC12510578; doi:10.1371/journal.pone.0332087)
Supplement: S2 Table — (DOCX) [file pone.0332087.s002.docx]

**S2 Table. Summary of domains and dimensions in the social determinants of health (SDH) questionnaire**

**Demographics (7 questions)***

Age, birthplace, citizenship, marital status, residence setting (e.g., urban/rural), sex, race

**Insurance, Access to Care (8 questions)**

Insurance status, type of insurance, recent history of routine and emergency medical care (location, frequency), has a regular doctor, limitations of seeing a doctor when desired

**Access and Adherence to Medicine and Therapies (7 questions)**

Prescription status, barriers to adherence to prescribed medicine, barriers to adherence, flu and

COVID-19 vaccine hesitancy, barriers to vaccination

**Lifestyle and Access to Necessities (11 questions)**

Access to food and fresh clean water, barriers of access to food and water, use of tobacco and alcohol, exercise, homelessness, housing arrangements (own, rent, living with others), feeling of safety in neighborhood, access to phone services, type of phone, car ownership, driver’s license

**Quality of Life (5 questions)**

Subjective general health, recent experience of physical health problems and impact on functioning and ability to work, recent experience of emotional health problems and impact on social functioning

**Social Support (7 questions)**

Availability of person(s) and family to provide companionship, comfort, and assistance when needed, feeling of safety in primary relationship(s)

**Medical History (16 questions)**

Cardiovascular, cancer, digestive, pulmonary, diabetes, arthritis, kidney, liver, anemia, dental

**Occupation, Education and Income (4 questions)**

Education level, employment status, job description, family income, dependents

*** Demographic factors were not included in the barriers to healthcare score.**
