# Supplementary material for: A social determinants of health survey in an Appalachian East Tennessee Medical Center: Initial findings and correlations with physical and emotional states of health
Source: PLoS One. 2025 Oct 9;20(10):e0332087. doi: 10.1371/journal.pone.0332087 (PMC12510578; doi:10.1371/journal.pone.0332087)
Supplement: S3 Table — (DOCX) [file pone.0332087.s003.docx]

**S3 Table. Some well-known previous attempts to develop and implement social determinants of health questionnaires**

| **First author, year, location** | **Population** | **SDH assessment instrument** | **Domains and dimensions of social determinants** | **Authors’ conclusions** |
| --- | --- | --- | --- | --- |
| Adler 2015, USA^39^ | No specific population | 22 SDH questions | Education, finances, psychological stress, depression, exercise, alcohol and tobacco consumption, social isolation, domestic violence | Including a concise panel of  standard measures of social and  behavioral determinants in every  patient’s EHR will increase clinical  awareness of the patient’s  health status and enable clinical,  public health, and community  resources to work in concert. |
| Page-Reeves, 2016, New Mexico, USA^36^ | Family Medicine clinics in New Mexico | “WellRx” questionnaire with 11 yes/no questions | Food, housing, access/cost of necessities, employment, income, education, drug or alcohol use by someone in home, personal safety, domestic abuse | The WellRx pilot demonstrated that it is feasible for a clinic to implement such an  assessment system, that the assessment can reveal important information, and that having information about patients’ social needs improves provider ease of practice. |
| Billioux, 2017, USA^29^ | No specific population | The Accountable Health Communities Health-Related Social Needs (AHC HRSN) Screening Tool with 10 core questions and 16 supplemental questions. | Core questions: Living situation, food, transportation, utilities, safety. Supplemental questions: Financial strain, employment, family and community support, education, physical activity, substance abuse, mental health, disabilities | The AHC HRSN screening tool was designed to identify the burden of unmet health-related social needs for several key non-medical drivers of health in a way that is broadly applicable across a spectrum of ages, conditions, backgrounds, and settings, while remaining streamlined enough to be incorporated into busy clinical workflows |
| Friedman, 2018, Oregon and Southwest  Washington, USA^37^ | 34 medical offices and 2 hospitals | Kaiser-Permanente developed 24 questions | Stress, lacking welfare support, lacking community resources, social isolation, legal issues, incarceration issues, financial, food, housing, access to necessities, can’t afford medicine, employment, income, fall risk, stressful work schedule, social environment, dental care, literacy, nutrition, exercise | The novel EHR-based tools developed by Kaiser-Permanente Northwest have led to use of standardized, measurable, and actionable SDH data to tailor and target specific resources to meet the identified needs of our patients. |
| Described in LaForge, 2018, USA^38^ | Kaiser-Permanente’s “Your Current Life Situation” | 32 SDH questions | Housing, finances, food insecurity, help with activities of daily living, marital/relationship status, education, access to medicine/supplies, social support/isolation, health literacy, psychological stress, domestic violence, access to dental care, health status, drug/alcohol use, illicit drug use | ---------- |
| Cottrell, 2019, USA^32^ | OCHIN clinical care sites in 20 U.S. states | “PRAPARE” questionnaire with 21 questions | Housing, education, employment, health insurance, income, food, access to care and necessities, transportation, social support, psychosocial stress, incarceration, refugee status, personal safety, domestic abuse | Simply activating EHR tools for SDH screening does not lead to widespread adoption of this practice. Potential barriers to screening adoption and implementation should be explored further. |
| Berkowitz, 2021, California, USA^40^ | Sutter Health | 11 SHD questions, pilot questionnaire | Alcohol consumption, finances, transportation, exercise, psychological stress, social isolation, domestic abuse and violence, depression, | This assessment of a pilot within a Sutter Health primary care clinic provides an  in-depth examination of an SDOH questionnaire and standard workflow intervention that can benefit dissemination within and outside of the Sutter Health network. |
| Oster, 2023, Australia^41^ | A convenience sample in Australia | Steps to Better Health questionnaire (STBH-Q), 19 SDH questions | Social isolation, employment, education, finances, deleterious habits or addictions, food insecurity, transportation, housing, mental health, physical health, access to recreation space, personal safety, access to health and legal services, quality of childhood | The STBH-Q explores multiple social determinants of health and has potential applicability for identifying need and linking clients to social support services in a range of settings, such as primary health care or via  social prescribing programs. |
| Health Leads, Boston, MA, USA^34^ | No specific population | Health Leads Social Needs Screening Tool with 10 yes/no questions | Food, housing, utilities, financial strain, transportation, childcare needs, literacy, social isolation, exposure to violence, urgency of needs |  |
| Health Begins, Pasadena, CA, USA^35^ | No specific population | Health Begins Upstream Screening Tool with 15 SDH questions | Education, employment, social isolation, physical activity, immigration, financial strain, housing, food, transportation, exposure to violence, stress, civic engagement | ---------- |
